# Positive and Negative Temperature Dependence in the Resistivity of Crystallized Zr-Fe-Ni Metallic Glasses

**DOI:** 10.3390/ma3125212

**Published:** 2010-12-07

**Authors:** Fathalla Hamed

**Affiliations:** Department of physics, Faculty of Science, United Arab Emirates University, Al-Ain, P.O. BOX 17551, UAE; E-Mail: Fhamed@uaeu.ac.ae; Tel.: +971-50-6631235; Fax: +971-3-7671291

**Keywords:** metallic glasses, amorphous alloys, electrical resistivity, recrystallization, nano-crystallization

## Abstract

Ni_0.25_Fe_0.75_Zr_3_ metallic glassy ribbons were annealed in evacuated quartz ampoules beyond the crystallization temperatures (T_x_ ~655 K) over the range 773 to 1,173 K for varying periods of time. The resistivity of samples annealed over the temperature range 923 to 1,073 K for periods less than four hours increased as a function of decreasing temperature, while it decreased for samples annealed for more than four hours or at temperatures below 923 K or above 1,073 K for any period of time. All the annealed samples were found to contain only Ni, Fe and Zr from energy dispersive X-ray (EDX) analyses.

## 1. Introduction 

Metallic glasses have become the subject of interest to a great number of scientists and technologists since they were first discovered in 1960 [1]. Mizutani has classified metallic glasses into five groups based on their electronic properties [2]. Non magnetic metallic glasses were classified as groups 4 and 5. Group 4 metallic glasses are defined as those for which the Fermi level is in the d band, while the Fermi level for group 5 is in the sp band. The temperature dependence of the electrical resistivity (ρ(T)) of group 5 metallic glasses over the temperature range 30–300 K can be understood within the framework of the generalized Faber-Ziman theory [2,3]. While for group 4 metallic glasses over the range 30–300 K, Mizutani proposed the following empirical equation ρ(T)/ρ(300 K) = A + B exp(−T/Δ), where A, B and Δ are fitting parameters [4]. At temperatures below 30 K, superconductivity and quantum interference effects arise [5,6]. Nano-crystallization of metallic glasses is an active field that promises materials with excellent physical properties [7,8,9]. Saida *et al*. found that icosahedral quasicrystalline particles ranging from 5 to 10 nm in diameter precipitated in Zr_70_Fe_20_Ni_10_ ternary metallic glass when it was annealed at 670 K for two minutes [10]. Altounian *et al.* found that the crystallization of FeZr_2_ metallic glass proceeds from the evolution of a large number of nano-crystallites 2.0–3.0 nm in size, and further annealing at 900 K for two hours resulted in grain growth of about 1 µm in size [11]. Liu *et al.* recently found that the crystallization of Fe_33_Zr_67_ and Fe_10_Zr_90_ is temperature and time dependent [12]. Upon the examination of the temperature dependence of the resistivity in FeZr_2_ metallic glass annealed at 900 K for a period of two hours, Dikeakos *et al.* found a negative temperature coefficient [13]. They have explained the observed behavior in terms of s-d scattering due to the presence of *cF*96 big cube structure. Negative temperature coefficients have also been observed in some ternary quasicrystals [14,15,16], in this case it was believed that the negative temperature coefficients are critically dependent on microstructure [17]. The purpose of the present study is to investigate the effect of annealing as a function of temperature and time on the temperature dependence of the resistivity in ternary Ni-Fe-Zr-based metallic glasses. We will present results that show positive and negative temperature dependence in fully crystallized metallic glassy alloys. 

## 2. Results and Discussion 

**Figure 1:**
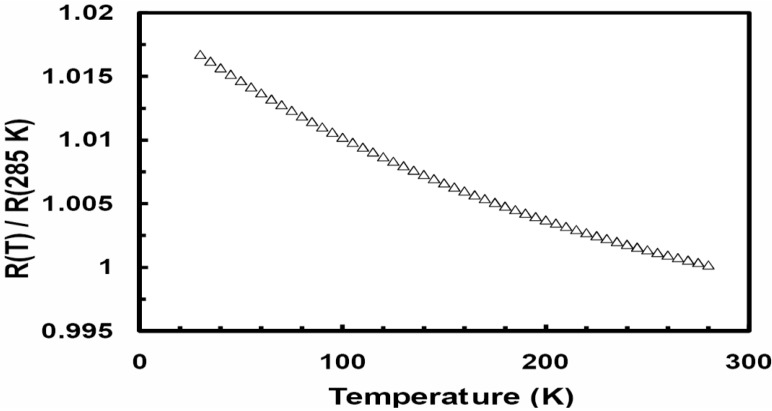
The temperature dependence of the relative electric resistivity (R(T)/R(285 K)) for the as quenched Ni_0.25_Fe_0.75_Zr_3_ metallic glass.

**Figure 2:**
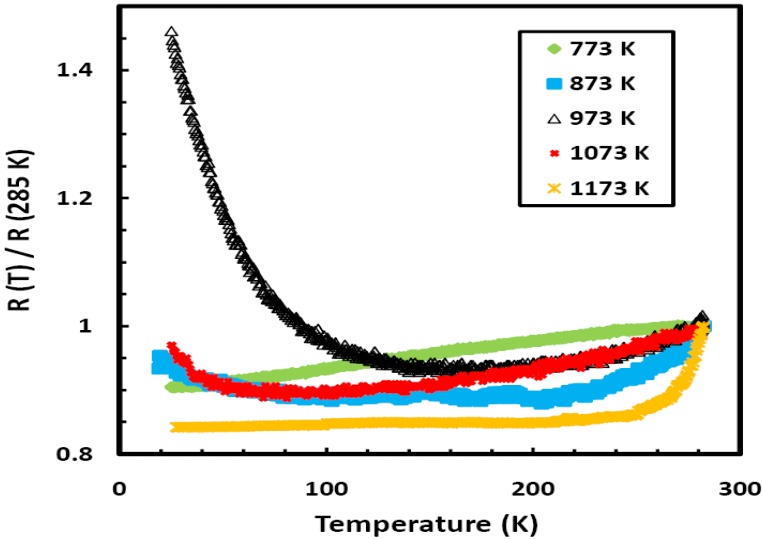
The temperature dependence of the relative resistivity (R(T)/R(285 K)) for different samples of Ni_0.25_Fe_0.75_Zr_3_ metallic glass annealed over the temperature range 773 to 1,173 K for the same period of one hour.

Here we present experimental results on the effect of the annealing on the temperature dependence of the electrical resistivity in Ni-Fe-Zr based metallic glasses. Figure 1 is a plot of the normalized resistivity (*ρ (T)*/*ρ* (285 K)) *versus* temperature for the as quenched Ni_0.25_Fe_0.75_Zr_3_ metallic glass. The amorphous state has a small negative temperature coefficient of resistivity; *α* (*α* = (1/*ρ)*∂*ρ/*∂*T*). The observed *ρ* (*T*) can be fitted to the following empirical equation proposed by Mizutani [2,3]: *ρ*(*T*)/*ρ*(300 K) = A + B *exp*(−*T*/Δ), where *ρ* (300 K) is the resistivity at 300 K and A, B and Δ are fitting parameters. This equation is considered for metallic glasses which contain d electrons at the Fermi energy level, E_F_. For the data fitted over the temperature range 30–300 K, A is approximately unity, B ~0.0287, and Δ = 235 K. Mizutani correlated Δ to the Debye temperature (θ_D_). The present Ni_0.25_Fe_0.75_Zr_3_ metallic glass is a group 4 metallic glass with the Fermi level in the d band. Figure 2 shows a plot of the relative electrical resistance (R(T)/R (285 K)) as a function of temperature for five different samples of Ni_0.25_Fe_0.75_Zr_3_ metallic glass annealed over the temperature range 773 to 1,173 K. All the samples were annealed inside evacuated sealed quartz ampoules for the same period of one hour. The crystallization temperature (T_x_) for the present metallic glass was determined from differential scanning calorimetry (DSC) analysis to be 655 K. The annealed samples were investigated using EDX analyses, all the samples were found to contain Ni, Fe, and Zr only and their compositions were very close to the composition of the un-annealed Ni_0.25_Fe_0.75_Zr_3_ metallic glass. Each of the samples used for the electrical measurements is approximately 1 cm long, ~1 mm wide and 20 μm thick; a measuring current of 1 mA was used. Figure 2 clearly shows the effect of annealing: there is pronounced effect for the sample annealed at 973 K. This sample shows negative temperature coefficient of resistivity (*α*), which is unusual for a metallic sample. We found that samples annealed over the temperature range 773 to 923 K, or above 1,073 K, for any period of time show positive temperature dependence, while samples annealed over the temperature range 923 to 1,073 K for less than four hours show clear signs of negative temperature dependence; however, the strongest effect is seen when the sample is annealed at 973 K. These results are unexpected since *ρ (T)* of metallic samples usually show positive temperature dependence. 

**Figure 3:**
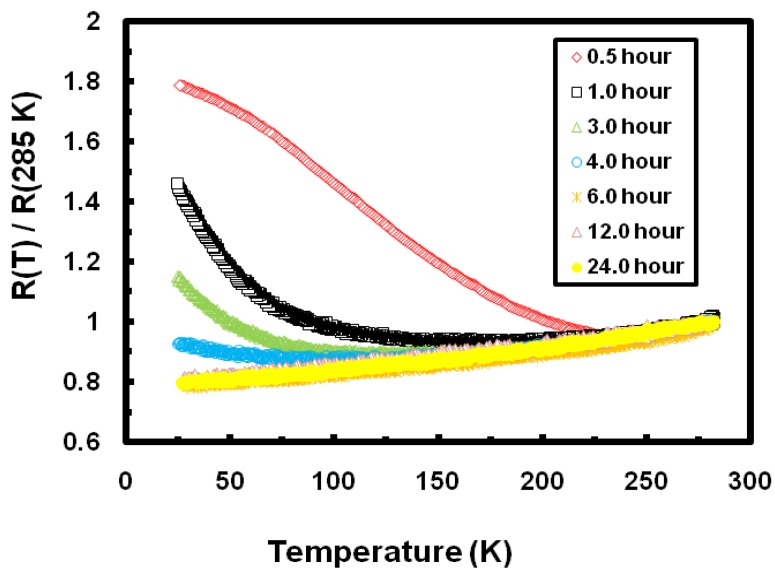
The temperature dependence of the relative electric resistivity (R(T)/R(285 K)) for eight different samples of Ni_0.25_Fe_0.75_Zr_3_ metallic glass annealed at 973 K for different periods of time.

To further investigate the nature of the observed negative temperature dependence and the effect of annealing, we concerted our efforts on annealing Ni_0.25_Fe_0.75_Zr_3_ metallic glasses at 973 K for varying periods of times. Figure 3 shows the effect of the period of annealing on *ρ (T)* for the 973 K annealed samples. As the annealing period is increased, the relative resistivity decreases and the negative temperature dependence becomes less pronounced. The samples annealed for periods of more than four hours show positive temperature dependence. The temperature dependence of the electrical resistivity of the 973 K annealed Ni_0.25_Fe_0.75_Zr_3_ metallic glass for half an hour is very similar to the result found by Dikeakos *et al* for FeZr_2_ metallic glass annealed at 900 K for two hours [13]. However, their study did not include any annealing for longer periods of time. For the observed results in the present study, we propose that the annealing of Ni_0.25_Fe_0.75_Zr_3_ metallic glass over the temperature range 923 to 1,073 K breaks the entire sample into a large number of nanocrystallites of different Ni, Ni-Zr, Fe, Fe-Zr and Ni-Fe-Zr phases. During the electrical measurements, the applied electric current has to tunnel through the barriers formed by the contact area between the surfaces of these nanocrystallites. As the temperature is lowered further below room temperature, the contact area between the nanocrystallites shrinks and the applied electric current finds it much harder to cross barriers, hence the observed negative temperature dependence. As the annealing period is increased, the nanocrystallites coalesce and form grains and the applied electric current then finds it much easier to cross the grain boundaries, hence the observed positive temperature dependence for samples annealed for more than four hours. 

**Figure 4:**
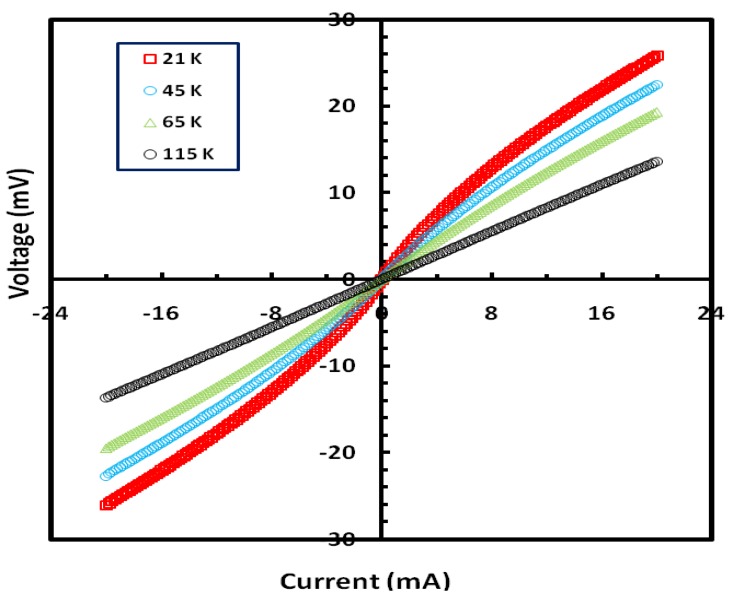
Current-Voltage (I-V) characteristics for a sample of Ni_0.25_Fe_0.75_Zr_3_ metallic glass annealed at 973 K for one hour.

To investigate the hypothesis of tunneling further, we carried out current-voltage (I-V) measurements at different temperatures. Figure 4 shows a plot of the *I-V* characteristics for a Ni_0.25_Fe_0.75_Zr_3_ metallic glass annealed at 973 K for one hour. The *I-V* curves display nonlinear behavior at low temperatures up to ~110 K, and the behavior becomes linear for temperatures above 110 K. The nonlinear behavior is consistent with our view that the applied current has tunnel through barriers formed at the contact area between the surfaces of the different nano-crystallites. This contact area decreases as the temperature is lowered. We should note here that all the observed effects in the present study were also measured in the presence of applied magnetic fields up to 3 KG but no noticeable differences were seen. 

**Figure 5:**
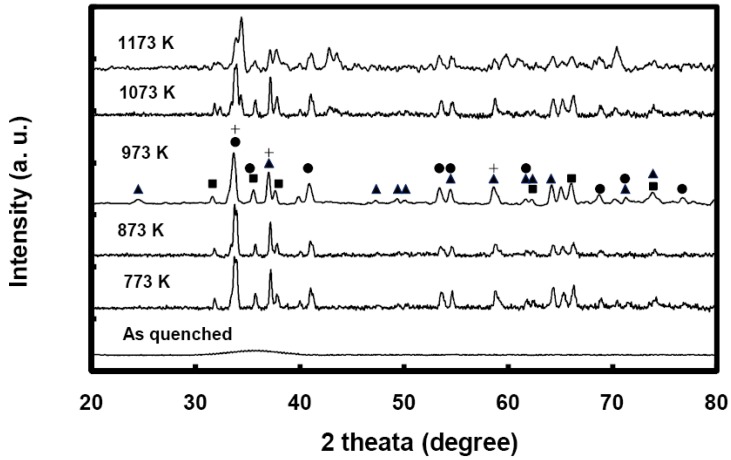
X-ray diffraction (XRD) profiles for different samples of Ni_0.25_Fe_0.75_Zr_3_ metallic glass annealed over the temperature range 773 to 1173 K for a period of one hour. (▲) fcc FeZr_2_, (■) bct FeZr_2_, (●) bct NiZr_2_, (+) Zr.

The crystallization kinetics in Zr-based metallic glasses is temperature and time dependent in a very complicated way [7,8,9]. The recent work of Liu *et al.* on the crystallization of Fe_33_Zr_67_ and Fe_10_Zr_90_ metallic glasses [12] is very relevant to the present study. They found that the crystallization of Fe_33_Zr_67_ and Fe_10_Zr_90_ metallic glasses over the temperature range 733–1,223 K proceeds by forming fine nanocrystallites of different Fe and FeZr phases, and the size of these fine grains grow as a function of annealing temperature and time. The crystallization of FeZr_2_ metallic glass proceeds by the formation of metastable fcc FeZr_2_ to fcc FeZr_2_ + bct FeZr_2_ and finally stable bct FeZr_2_ [12], while the crystallization of NiZr_2_ metallic glass forms a stable bct NiZr_2_ [11]. We carried out X-ray diffraction (XRD) studies to determine the nature of our annealed metallic glasses. Figure 5 shows the XRD profiles for different samples of Ni_0.25_Fe_0.75_Zr_3_ metallic glasses annealed at different temperatures for one hour. It is very clear that the annealed samples are fully crystallized. The broadening of the diffraction peaks is an indication of fine grains and nanocrystallites. In accordance with the Scherrer formula, the grain sizes would vary anywhere between 30 and 70 nm. This suggests that the fully crystallized metallic glasses are composed entirely of a large number of nanocrystallites of different phases containing Fe, Ni and Zr. The diffraction peaks for the 973 K annealed metallic glass are identified with NiZr_2_ and FeZr_2_ crystalline phases. In Figure 5, the triangles represent metastable fcc FeZr_2_, squares represent stable bct FeZr_2_, circles represent stable bct NiZr_2_, and crosses represent hcp Zr. In comparison to the XRD profile of the 973 K annealed sample, the diffraction peaks for the 773 K, 873 K, and 1,073 K annealed samples are about 10–15% narrower and contain no or minute amounts of fcc FeZr_2_.

We believe that the annealed samples break into a large number of different crystallites of different phases all competing with each other as a function of temperature and annealing time. In the case of the 973 K annealed samples, the nanocrystallites coalesce into much bigger grains as the time of annealing is increased and some phases may disappear in favor of others; such as the gradual disappearance of the metastable fcc FeZr_2_. The fcc FeZr_2_ phase is a metastable phase and it is also known as the big cube structure with 96 atoms. Dikeakos *et al.* have examined the temperature dependence of the resistivity of the big cube phase over the temperature range 1.6–300 K [13]. Their examination revealed a large negative temperature coefficient (α = (1/ρ)/(dρ/dT)). We believe the observed negative temperature dependence in our samples is due to the presence of metastable phases of fcc FeZr_2_ or even fcc NiZr_2_ combined with tunneling effects. As the period of the annealing is increased, the metastable phases transform into more stable phases such as bct FeZr_2_ and NiZr_2_, adding to this the coalescence of the fine nanocrystallites into much bigger grains. Subsequently, the tunneling effects and the metastable phases disappear and we observe positive temperature dependence. 

## 3. Experimental Section 

The Zr-Ni-Fe alloy ingots were prepared by arc-melting appropriate amounts (75% Zr, 6.25% Ni, and 18.75% Fe) of high purity (99.99%) constituent elements in a water-cooled copper hearth with Zirconium getter in an argon atmosphere. The resultant ingots (1.5 grams) were melt-spun under 40 Kpa Ar atmosphere on the surface of a solid 4-inch copper wheel rotating with tangential speed of 35 m/s. The resultant ribbons were typically 1 m long, ~1 mm wide and 20 μm thick. The amorphous nature of the obtained ribbons was studied by Cu Kα X-ray diffraction. The ribbons were deemed to be amorphous based on the absence of diffraction peaks. A Jeol JSM-5600 scanning electron microscope equipped with Oxford EDX was used to determine the morphology, elemental composition and homogeneity of the produced ribbon. Differential scanning calorimetry (DSC) analyses were carried out in a Perkin-Elmer DSC7 under Helium atmosphere at a heating rate of 20 K/min. 5 to 7 cm long ribbons of Ni_0.25_Fe_0.75_Zr_3_ metallic glass were sealed inside a quartz ampoule under vacuum of about 10^-5^ torr. This step was taken to avoid any oxidation of the metallic glass during the heating treatment. The sealed ampoule was then placed inside a Thermolyne 1300 furnace where the desired constant annealing temperature can be set. Different samples of the Ni_0.25_Fe_0.75_Zr_3_ metallic glasses sealed inside quartz ampoules were annealed over the temperature range 773 to 1,173 K for different periods of time. After the annealing process was finished, the quartz ampoules were allowed to cool, then were broken and the annealed samples were collected for further studies. A 4-point dc resistivity technique was employed in the measurement of the electrical resistance. A constant current was applied by a programmable 236 Keithley source measure unit. The current direction was switched from positive to negative for each measurement and the magnitudes of the positive and negative voltage readings were averaged in order to eliminate any possible thermocouple voltage induced at the electrical leads.

## 4. Conclusions 

Ni-Fe-Zr metallic glasses annealed over the temperature range 923 to 1,073 K for periods of less than four hours produced negative temperature dependence at low temperatures. Alternatively, annealing for more than four hours over the same temperature range produced positive temperature dependence. We have found that annealing at 973 K produced metallic alloys with the strongest negative temperature. 
